# The influence of postoperative complications on long-term prognosis in patients with colorectal carcinoma

**DOI:** 10.1007/s00384-020-03557-3

**Published:** 2020-03-14

**Authors:** Clemens Beck, Klaus Weber, Maximilian Brunner, Abbas Agaimy, Sabine Semrau, Robert Grützmann, Vera Schellerer, Susanne Merkel

**Affiliations:** 1 Department of Surgery, Friedrich-Alexander-Universität Erlangen-Nürnberg, Erlangen, Germany; 2 Institute of Pathology, Friedrich-Alexander-Universität Erlangen-Nürnberg, Erlangen, Germany; 3 Department of Radiation Oncology, Friedrich-Alexander-Universität Erlangen-Nürnberg, Erlangen, Germany

**Keywords:** Colorectal carcinoma, Postoperative complications, Clavien-Dindo classification, Prognosis

## Abstract

**Background:**

The impact of postoperative complications (POCs) on the long-term prognosis of patients with colorectal carcinoma was analysed with respect to their severity according to the Clavien-Dindo classification (CDC).

**Methods:**

The prospectively collected data of 2158 patients who underwent curative resection of a colorectal carcinoma (1168 rectal carcinomas, 990 colon carcinomas) without distant metastases from 1995 to 2014 were analysed. The POCs were documented in a standardized form and graded with the CDC. Patients who died postoperatively (CDC grade V, 1.7%) were excluded.

**Results:**

In total, 467 patients (21.6%) had POCs: CDC I, 141 (6.5%); CDC II, 162 (7.5%); CDC III, 112 (5.2%); and CDC IV, 52 (2.4%). More POCs and higher CDC grades were found in men, ASA III-IV patients, rectal carcinoma patients, and patients who underwent abdominoperineal excisions or multivisceral resections. The 5-year locoregional recurrence rate was 5.3% in patients without POCs and 6.6% in patients with POCs. It was highest in CDC III patients (12.9%), which was confirmed in multivariate analysis (HR 2.2; *p* = 0.005). The 5-year distant metastasis rate was 15.9% in CDC 0 patients and 19.5% in CDC I–IV patients. In multivariate analysis, distant metastasis was highest in CDC III patients (HR 1.7; *p* = 0.020). The 5-year overall survival rate was 83.5% in patients without POCs and 73.5% in patients with POCs. It was worst in CDC IV patients (63.1%), which was confirmed by multivariate analysis (HR 1.9; *p* = 0.001).

**Conclusion:**

Patients with POCs after colorectal surgery have a poor long-term prognosis. As the CDC grade increases, survival deteriorates.

## Introduction

Quality management in colorectal carcinoma is usually divided into short- and long-term results. How closely these two are linked was first demonstrated with anastomotic leaks. In 1991 Akyol et al. [1] and in 2001 Merkel et al. [2] showed that anastomotic leaks in rectal carcinoma, a traditional short-term quality indicator, was associated with a poor long-term outcome, i.e., elevated locoregional recurrence rates and poor survival. Moreover, this has also been proven in meta-analyses [3]. Over the last few decades, the frequency of anastomotic leaks has decreased due to improvements in surgical techniques. Whether there is a connection between postoperative complications (POCs) in general and long-term prognosis is still under debate, particularly whether a classification of complications can gradually predict the long-term outcome of patients with colorectal cancer.

In 1992, Clavien et al. [4] classified negative outcomes by differentiating complications, sequelae, and failures. Twelve years later, the classification was re-evaluated and modified by one of the authors, focusing on the grading of life-threatening complications and long-term disabilities due to complications [5]. The use of therapeutic consequences as a basis for the classification of complications remained unchanged. Although new attempts to classify complications appeared, the Clavien-Dindo classification (CDC) has been validated and established in international studies across many fields of surgery. Recently, a study of 2266 patients reported that POCs are associated with adverse oncological outcomes, with an increased effect at higher CDC grades [6].

The Erlangen Registry for Colorectal Carcinoma (ERCRC) was established in 1978. Quality management and prognostic factor analysis are the main objectives of this prospective cancer registry. The aim of the current study was to analyse the impact of postoperative complications on long-term prognosis and to assess the severity of complications graded by the CDC and their influence on locoregional recurrence, distant metastasis, disease-free survival, and overall survival.

## Methods

The study refers to 2215 consecutive patients who underwent colorectal carcinoma surgery at the Department of Surgery of the University Hospital Erlangen, Germany, between 1995 and 2014. We evaluated the prospectively collected database of the Erlangen Registry for Colorectal Carcinomas (ERCRC) according to the following inclusion criteria: solitary invasive colorectal carcinoma (at least into the submucosa); no appendiceal carcinoma; no other previous or synchronous malignancies; carcinomas not related with familial adenomatous polyposis, ulcerative colitis or Crohn’s disease; no distant metastases; radical elective surgery; and residual tumour classification R0 (no residual tumour at clinical and pathohistological examination). All patients underwent resection with regional lymph node dissection according to the standards of total or partial mesorectal excision (TME, PME) [7] or complete mesocolic excision (CME) [8]. Fifty-seven patients had to be excluded: 37 patients (1.7%) who died postoperatively (Clavien-Dindo classification V) and 20 patients (0.9%) with unknown follow-up information. Finally, we included 2158 patients in the analysis.

Carcinomas with a distal margin ≤ 16 cm to the anal verge were classified as rectal carcinomas and those with a distal margin > 16 cm were classified as colon carcinomas [9].

After preoperative staging, long-term preoperative chemoradiation was administered for locally advanced rectal carcinomas (cT3,4 or cN+) and for selected lower-third carcinomas (< 6 cm) to enable sphincter preservation. Of the 545 patients (46.7%) with neoadjuvant therapy, the majority of the patients (*n* = 250) received chemoradiation according to the protocol presented by the German CAO/ARO/AIO-94 study [10], with radiotherapy consisting of a total of 50.4 Gy apportioned in 28 fractions of 1.8 Gy, five times weekly and a continuous 120-h infusion of 5-fluorouracil with a dose of 1000 mg/m^2^ per day during the first and fifth week. A total of 91 patients received oral capecitabine instead of an infusion of 5-FU. In addition, 235 patients received oxaliplatin, 17 patients received irinotecan, and 8 patients received additional cetuximab. During the last few years, hyperthermia was provided to some patients within clinical trials. Six to eight weeks after the completion of preoperative chemoradiation, the patients underwent open TME surgery [7] or PME surgery in selected patients with carcinomas of the upper third (12–16 cm).

Patients with colon carcinomas were treated by CME. The extent of surgery was always determined by the localization of the tumour and its potential lymphatic spread. Carcinomas of the transverse colon, hepatic or splenic flexure were treated with extended hemicolectomies or subtotal colectomies. Adjuvant chemotherapy was recommended for stage III patients and for select stage II patients.

The detailed documentation allowed for a classification of the carcinomas according to the eighth edition of the Tumour Node Metastasis (TNM) classification system [11].

Postoperative complications (POCs) were documented in a standardized form. To assess the severity of these POCs, the Clavien-Dindo classification (CDC) [5, 12] was retrospectively applied. This classification consists of 5 different grades. Grade I comprises any deviation from the normal postoperative course without the need for pharmacological treatment or surgical, endoscopic and radiological interventions. The allowed therapeutic regimens are antiemetics, antipyretics, analgesics, diuretics, electrolytes and physiotherapy. This grade also includes wound infections opened at the bedside. Grade II complications require pharmacological treatment with drugs other than those applied for grade I complications, including blood transfusions and total parenteral nutrition. Grade III complications require surgical, endoscopic or radiological intervention (grade III a, intervention not under general anaesthesia; grade III b, intervention under general anaesthesia). Grade IV complications are life-threatening complications requiring intensive care/intermediate care management (grade IV a, single organ dysfunction including dialysis; grade IV b, multi-organ dysfunction). Grade V complications describe the postoperative death of the patient.

Anastomotic leaks were classified according to Rahbari et al. (grade A, no change in patients’ management; grade B, active therapeutic intervention without relaparotomy; grade C, requiring re-laparotomy [13]). Severe late anastomotic leaks diagnosed after readmission were also considered.

The American Society of Anaesthesiologists (ASA) classifies the comorbidities of the patients into 6 levels [14]. The levels were grouped into ASA I-II and ASA III-IV. The ASA classification was missing in 324 patients.

The patients were followed up for at least 5 years, every 3 months for the first 2 years and every 6 months thereafter; the follow-up included a physical examination, carcinoembryonic antigen (CEA) analysis, abdominal ultrasonography, chest X-ray, computed tomography (CT) of the pelvis and rectoscopy/colonoscopy, depending on localization (rectum or colon) and stage of the primary tumour. In 2004, the follow-up examinations were changed to semi-annually for the first 2 years and then yearly for a total of 5 years according to the first edition of the German S3-Guidelines for Colorectal Carcinoma [15]. A locoregional recurrence was defined as the presence of a recurrent tumour located at the anastomosis, in the tumour bed or in residual lymph nodes, as diagnosed by a clinical and/or pathological examination. Distant metastases were typically diagnosed using imaging techniques; histological confirmation was encouraged. Follow-up data were collected either at the university hospital or from written correspondence with the patients’ family doctors. Thereafter, at least the vital status of each patient was regularly monitored through inquiries at the patients` local registration office. The median follow-up time of all patients was 9 years (0–23 years).

## Statistical analysis

The χ^2^ test and Fisher’s exact test were used to compare frequencies, and the Mann-Whitney U test was used to analyse continuous data. The Kaplan-Meier method was used to calculate the 5-year rates of locoregional recurrence, distant metastasis and survival. Survival curves were compared using a log-rank test. Disease-free survival was defined as the time to the first occurrence of locoregional recurrence, distant metastasis or death by any cause. The endpoint of overall survival was death from any cause. Factors that were found to be significant in the univariate analysis were included in a multivariate Cox regression model. A *p* value less than 0.05 was considered significant. Statistical analysis was carried out using the statistics software package SPSS® version 21 (IBM, Armonk, New York, USA).

## Results

The characteristics of all 2158 patients and their tumours are shown in Table 1. A total of 467 (21.6%) patients had postoperative complications. These complications were distributed across the 4 CDC grades as follows: grade I, *n* = 141 (6.5%); grade II, *n* = 162 (7.5%); grade III, *n* = 112 (5.1%); and grade IV, *n* = 52 (2.4%). Table 2 shows typical examples of postoperative complications and their respective treatment according to the different CDC grades. The most frequent complications were: for CDC grade I complications, secondary healing (*n* = 28) and urinary bladder dysfunction (*n* = 40); for CDC grade II complications, urinary tract infection (*n* = 40) and central venous catheter infection (*n* = 23); for CDC grade III, anastomotic leak (*n* = 27), abdominal wall dehiscence (*n* = 14) and postoperative bleeding (*n* = 11); and for CDC grade IV, anastomotic leak (*n* = 23) and pulmonary complication (*n* = 12). Compared to other patients, male patients, patients with an ASA level > II, tumour localization in the rectum, abdominoperineal excision and multivisceral resection had significantly more postoperative complications and higher CDC grades.

**Table 1:** Patients’ and tumour characteristics for 2158 patients

Clavien-Dindo classification	All *n* = 2158	0 *n* = 1691	I *n* = 141	II *n* = 162	III *n* = 112	IV *n* = 52	*p*
	*N* (%)	*n* (%)	*n* (%)	*n* (%)	*n* (%)	*n* (%)	
Age median (range) (years)	64.5 (17–94)	64 (17–94)	66 (21–87)	67 (36–93)	65 (35–88)	71 (39–90)	< 0.001
Sex							
Male	1351 (62.6)	1038 (61.4)	92 (65.2)	99 (61.1)	87 (77.7)	35 (67)	
Female	807 (37.4)	653 (38.6)	49 (34.8)	63 (38.9)	25 (22.3)	17 (33)	0.011
ASA*							
ASA I–II	1498 (81.7)	1200 (83.7)	93 (77.5)	114 (78.6)	65 (69.9)	26 (61)	
ASA III–IV	336 (18.3)	233 (16.3)	27 (22.5)	31 (21.4)	28 (30.1)	17 (40)	< 0.001
Tumour site							
Colon	990 (45.9)	807 (47.7)	61 (43.3)	62 (38.3)	33 (29.5)	27 (52)	
Rectum	1168 (54.1)	884 (52.3)	80 (56.7)	100 (61.7)	79 (70.5)	25 (48)	< 0.001
Surgical procedure							
(Low) anterior resection	951 (44.1)	747 (44.2)	48 (34.0)	77 (47.5)	57 (50.9)	22 (42)	
Abdominoperineal excision	216 (10.0)	138 (8.2)	30 (21.3)	23 (14.2)	22 (19.6)	3 (6)	
Colon standard resection	787 (36.5)	656 (38.8)	40 (28.4)	50 (30.9)	26 (23.2)	15 (29)	
Colon extended resection	204 (9.5)	150 (8.9)	23 (16.3)	12 (7.4)	7 (6.3)	12 (23)	< 0.001
Multivisceral resection	246 (11.4)	170 (10.1)	26 (18.4)	23 (14.2)	17 (15.2)	10 (19)	0.003
Multimodal treatment							
Neoadjuvant treatment	550 (25.5)	420 (24.8)	37 (26.2)	43 (26.5)	39 (34.8)	11 (21.2)	0.187
Adjuvant treatment	658 (30.5)	537 (31.8)	44 (31.2)	41 (25.3)	28 (25.0)	8 (15.4)	0.032
Adjuvant treatment for rectal carcinoma stage II,III	162/342 (47.4)	126/253 (49.8)	10/29 (34)	11/34 (32)	10/17 (59)	5/9 (56)	0.150
Adjuvant treatment for colon carcinoma stage III	215/312 (68.9)	186/257 (72.4)	14/17 (82)	10/15 (67)	5/12 (42)	0/11 (0)	< 0.001
pT category							
pT1,2/ypT0,1,2	998 (46.2)	790 (46.7)	53 (37.6)	76 (46.9)	59 (52.7)	20 (38)	
pT3/ypT3	1028 (47.6)	801 (47.4)	80 (56.7)	74 (45.7)	45 (40.2)	28 (54)	
pT4/ypT4	132 (6.1)	100 (5.9)	8 (5.7)	12 (7.4)	8 (7.1)	4 (8)	0.309
pN category							
pN0/ypN0	1491 (69.1)	1166 (69.0)	95 (67.4)	119 (73.5)	81 (72.3)	30 (58)	
pN1,2/ypN1,2	667 (30.9)	525 (31.0)	46 (32.6)	43 (26.5)	31 (27.7)	22 (42)	0.290
Stage (UICC)							
Stage I	573 (26.6)	454 (26.8)	32 (22.7)	42 (25.9)	33 (29.5)	12 (23)	
Stage II	535 (24.8)	424 (25.1)	37 (26.2)	46 (28.4)	17 (15.2)	11 (21)	
Stage III	500 (23.2)	393 (23.2)	35 (24.8)	31 (19.1)	23 (20.5)	18 (35)	
Stage y0	73 (3.4)	60 (3.5)	2 (1.4)	4 (2.5)	7 (6.3)	0	
Stage yI	164 (7.6)	127 (7.5)	6 (4.3)	17 (10.5)	12 (10.7)	2 (4)	
Stage yII	146 (6.8)	101 (6.0)	18 (12.8)	10 (6.2)	12 (10.7)	5 (10)	
Stage yIII	167 (7.7)	132 (7.8)	11 (7.8)	12 (7.4)	8 (7.1)	4 (8)	0.045

*ASA* American Society of Anesthesiologists Classification; *ASA missing in 324 patients

**Table 2:** Examples of postoperative complications and treatment according to different CDC grades

Postoperative complication	CDC I	CDC II	CDC III	CDC IV
Pulmonary complication	Mild infection requiring respiratory therapy	Pneumonia requiring antibiotics	Pleural effusion requiring drainage	Respiratory insufficiency requiring ventilation
Urologic complication	Urinary bladder dysfunction requiring leg bags or sanitary pads	Urinary tract infection requiring antibiotics; bladder dysfunction treated by anticholinergics or alpha-blocking agents	Urinary bladder dysfunction treated by suprapubic catheterization	Urosepsis requiring intensive care
Wound healing disorder	Wound infection opened at the bedside	Phlegmonous wound infection requiring antibiotics	Wound dehiscence requiring surgical abdominal wall closure	Wound infection leading to sepsis requiring intermediate or intensive care
Anastomotic leak	Anastomotic leak requiring observation	Anastomotic leak requiring antibiotics	Anastomotic leak requiring drainage or relaparatomy	Major leak with peritonitis and multi-organ dysfunction requiring relaparotomy and intensive care

## Quality indicators

The median number of regional lymph nodes examined in all specimens was 25 (1–145). In patients with rectal carcinoma, the median number of regional lymph nodes examined was 22 (2–76). In patients with primary surgery, it was 27 (4–76), and in those with neoadjuvant treatment, it was 18 (2–62). In patients with colon carcinoma, the median number of regional lymph nodes examined was 29 (1–145). In those who underwent standard colon resections, it was 27 (4–76), and in extended resections, it was 37 (12–145).

The pathological circumferential resection margin (pCRM) was positive (≤ 1 mm) in 20 (2.0%) of the 997 rectal cancer specimens who underwent respective examinations.

The quality of TME/PME [16] was evaluated as mesorectal plane or intramesorectal plane of surgery in 911 (98.3%) of 927 specimens, while 16 (1.7%) specimens were classified as muscularis propria plane.

Anastomotic leaks of grade B and C were observed in 45 (4.8%) of 942 patients after rectal resection and in 23 (2.3%) of 985 patients after colon resection.

## Adjuvant treatment

Six hundred fifty-eight patients (30.5%) received adjuvant treatment. The higher the CDC grade, the less frequently adjuvant therapy was administered (31.8% to 15.4%; Table 1). While this was not observed for stage II and III rectal cancer patients without neoadjuvant therapy, it was highly significant for stage III colon carcinomas (*p* < 0.001).

## Prognosis

**Fig. 1:**
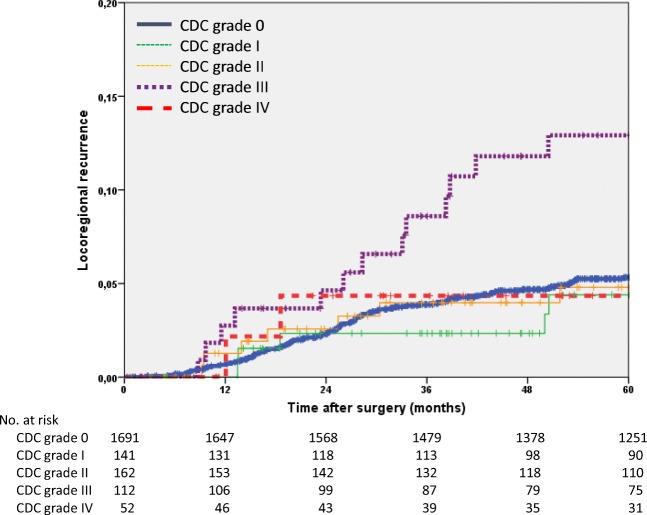
Kaplan-Meier curves of the time to locoregional recurrence (*n* = 2158)

The 5-year locoregional recurrence rate of the entire study group was 5.6% (95% confidence interval 4.6–6.6). The rate was 8.5% for rectal carcinomas compared to 2.2% for colon carcinomas (*p* < 0.001). The locoregional recurrence rate was significantly higher in men than in women and increased with stage. The rate was higher in patients with POCs (6.6% vs 5.3%; *p* = 0.068) and was highest in patients with CDC grade III POCs (12.9%; *p* = 0.007). In the multivariate Cox regression analysis, sex (*p* = 0.050), tumour site (*p* < 0.001), stage III tumours (*p* = 0.005) and CDC grade III POCs were found to significantly influence the locoregional recurrence rate (HR 2.2; *p* = 0.005; Table 3, Fig. 1).

**Table 3:** Univariate and multivariate Cox regression analyses of locoregional recurrences

		Univariate analysis			Multivariate model		
	*n*	5-year rate	95%CI	p**	Hazard ratio	95% CI	*p*
All	2158	5.6	4.6–6.6				
Age (years)							
< 65	1079	5.7	4.3–7.1				
≥ 65	1079	5.6	4.2–7.0	0.718			
Sex							
Male	1351	6.5	5.1–7.9		1.0		
Female	807	4.1	2.7–5.5	0.010	0.7	0.4–1.0	0.050
ASA*							
ASA I–II	1498	4.8	3.6–6.0				
ASA III–IV	336	6.4	3.5–9.3	0.344			
Tumour site							
Colon	990	2.2	1.2–3.2		1.0		
Rectum	1168	8.5	6.7–10.3	< 0.001	5.3	3.2–8.8	< 0.001
Stage (UICC)							
Stage I	573	3.5	1.9–5.1		1.0		
Stage II	535	4.6	2.8–6.4		2.2	1.3–4.0	0.006
Stage III	500	7.7	5.2–10.2		2.7	1.5–4.6	< 0.001
Stage y0	73	0			0		0.954
Stage yI	164	4.5	1.2–7.8		0.6	0.3–1.5	0.296
Stage yII	146	9.6	4.5–14.7		1.7	0.9–3.3	0.118
Stage yIII	167	10.2	5.3–15.1	< 0.001	1.9	1.0–3.5	0.061
Clavien-Dindo classification							
0	1691	5.3	4.1–6.5		1.0		
I	141	4.4	0.7–8.1		0.9	0.4–1.8	0.683
II	162	4.8	1.3–8.3		1.0	0.5–1.9	0.948
III	112	12.9	6.2–19.6		2.2	1.3–3.9	0.005
IV	52	4.3	0–10.2	0.007	0.8	0.2–3.1	0.699

*ASA* American Society of Anesthesiologists Classification; *ASA missing in 324 patients

**Log-rank test

**Fig. 2:**
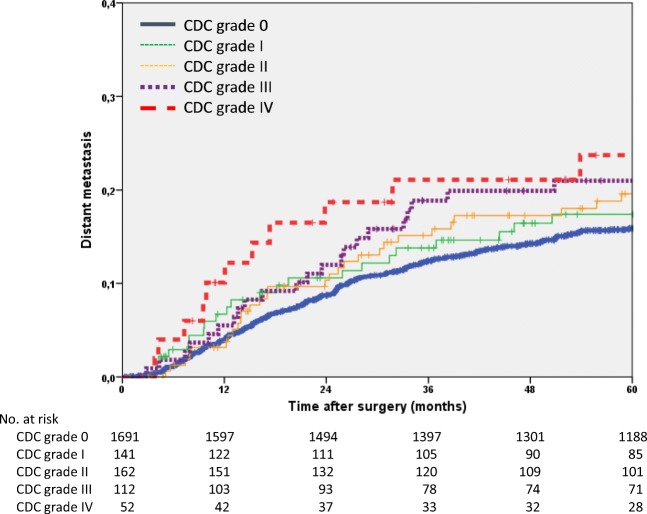
Kaplan-Meier curves of the time to distant metastases (*n* = 2158)

The 5-year distant metastasis rate of the entire study group was 16.7% (95% CI 15.1–18.3). The rate differed significantly between patients with and without POCs (19.5% vs 15.9%; *p* = 0.009). The univariate analysis also found that ASA classification (*p* = 0.028), tumour site (*p* < 0.001) and tumour stage (*p* < 0.001) were significant factors. The 5-year distant metastasis rate increased as the CDC grade advanced, but this association did not reach significance (*p* = 0.066). In the multivariate analysis, ASA classification (*p* = 0.045), tumour site (*p* < 0.001) and stage III tumours (*p* = 0.020) were found to be independent prognostic factors. In addition, CDC grade III POCs were again significantly associated with the highest risk for distant metastases (HR 1.7; *p* = 0.020; Table 4, Fig. 2).

**Table 4:** Univariate and multivariate Cox regression analyses of distant metastases

		Univariate analysis			Multivariate model		
	n	5-year rate	95% CI	p**	Hazard ratio	95% CI	*p*
All	2158	16.7	15.1–18.3				
Age (years)							
< 65	1079	16.5	14.3–18.7				
≥ 65	1079	16.9	14.5–19.3	0.598			
Sex							
Male	1351	17.9	15.7–20.1				
Female	807	14.6	12.1–17.1	0.051			
ASA*							
ASA I-II	1498	15.5	13.5–17.5		1.0		
ASA III-IV	336	20.0	15.3–24.7	0.028	1.3	1.0–1.8	0.045
Tumour site							
Colon	990	12.9	10.7–15.1		1.0		
Rectum	1168	19.9	17.5–22.3	< 0.001	1.8	1.4–2.4	< 0.001
Stage (UICC)							
Stage I	573	6.3	4.1–8.5		1.0		
Stage II	535	13.1	10.2–16.0		2.5	1.7–3.8	< 0.001
Stage III	500	28.8	24.7–32.9		4.7	3.3–6.9	< 0.001
Stage y0	73	1.4	0–4.1		0.1	0.0–1.0	0.047
Stage yI	164	8.8	4.5–13.1		1.0	0.6–1.8	0.941
Stage yII	146	14.8	7.7–21.9		3.3	2.1–5.2	< 0.001
Stage yIII	167	36.4	29.0–43.8	< 0.001	4.8	3.2–7.4	< 0.001
Clavien-Dindo classification							
0	1691	15.9	14.1–17.7		1.0		
I	141	17.4	10.7–24.1		1.0	0.7–1.6	0.878
II	162	19.6	13.1–26.1		1.4	1.0–2.1	0.056
III	112	21.0	13.2–28.8		1.7	1.1–2.5	0.020
IV	52	23.7	11.4–36.0	0.066	1.2	0.6–2.5	0.543

*ASA* American Society of Anesthesiologists Classification; *ASA missing in 324 patients. **Log-rank test

**Fig. 3:**
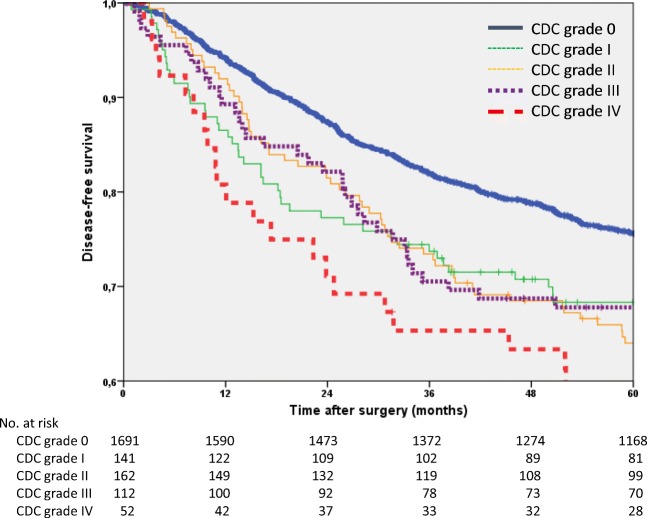
Kaplan-Meier curves of disease-free survival (*n* = 2158)

The 5-year disease-free survival rate of the entire study group was 73.4% (95% CI 71.4–75.4). The patients with POCs had a significantly worse disease-free survival (65.2%) than patients without POCs (75.6%; *p* < 0.001). In the univariate analysis, significant differences were found for age, sex, ASA classification, tumour site, tumour stage and CDC grade. In the multivariate analysis adjusted for age, all of these factors were found to have a significant influence on disease-free survival. Patients with CDC grade IV POCs were found to have the worst disease-free survival compared to patients without POCs (HR 1.8; *p* = 0.002; Table 5, Fig. 3).

**Table 5:** Univariate and multivariate Cox regression analyses of disease-free survival

		Univariate analysis			Multivariate model adjusted for age		
	*n*	5-year rate	95% CI	p**	Hazard ratio	95% CI	*p*
All	2158	73.4	71.4–75.4				
Age (years)							
< 65	1079	78.7	76.2–81.2				
≥ 65	1079	68.0	65.3–70.7	< 0.001			
Sex							
Male	1351	71.6	69.2–74.0		1.0		
Female	807	76.3	73.4–79.2	0.001	0.8	0.7–1.0	0.015
ASA*							
ASA I-II	1498	77.4	75.2–79.6		1.0		
ASA III-IV	336	57.1	51.8–62.4	< 0.001	1.8	1.5–2.1	< 0.001
Tumour site							
Colon	990	76.8	74.1–79.5		1.0		
Rectum	1168	70.5	68.0–73.0	0.047	1.4	1.2–1.7	< 0.001
Stage (UICC)							
Stage I	573	83.8	80.7–86.9		1.0		
Stage II	535	74.4	70.7–78.1		1.4	1.1–1.7	0.001
Stage III	500	61.0	56.7–65.3		1.9	1.5–2.3	< 0.001
Stage y0	73	97.3	93.6–100		0.6	0.3–1.0	0.047
Stage yI	164	85.7	80.2–91.2		0.8	0.6–1.1	0.171
Stage yII	146	65.5	57.7–73.3		1.6	1.2–2.1	0.002
Stage yIII	167	56.2	48.6–63.8	<0.001	2.1	1.6–2.8	< 0.001
Clavien-Dindo classification							
0	1691	75.6	73.4–77.8		1.0		
I	141	68.3	60.5–76.1		1.5	1.2–2.0	0.002
II	162	64.0	56.6–71.4		1.4	1.1–1.8	0.002
III	112	67.8	59.2–76.4		1.4	1.1–1.9	0.013
IV	52	55.4	41.9–68.9	<0.001	1.8	1.2–2.5	0.002

*ASA* American Society of Anesthesiologists Classification; *ASA missing in 324 patients. **Log-rank test

**Fig. 4:**
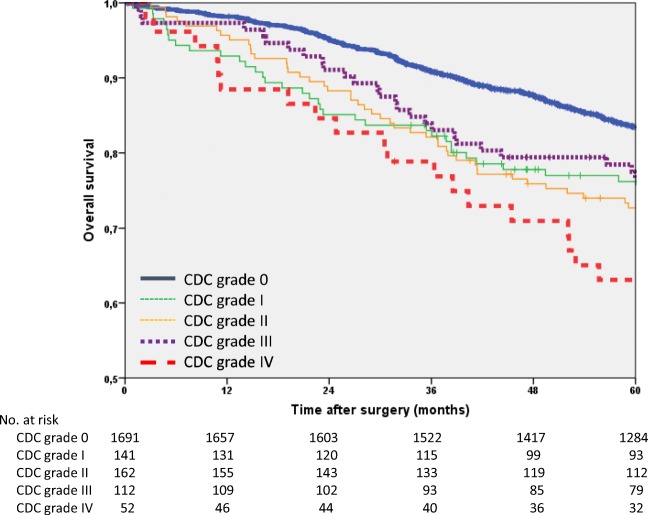
Kaplan-Meier curves of overall survival (*n* = 2158)

The 5-year overall survival rate of the entire study group was 81.3% (95% CI 79.7–82.9). The patients with POCs had a strikingly decreased overall survival (73.5%) compared to patients without POCs (83.5% *p* < 0.001). The univariate analysis was significant for age (*p* < 0.001), sex (*p* = 0.001), tumour stage (*p* < 0.001) and CDC grade (*p* < 0.001). In the multivariate analysis, all of these factors were found to be significant. Patients with CDC grade IV POCs had the worst overall survival (HR 1.9; *p* = 0.001; Table 6, Fig. 4).

**Table 6:** Univariate and multivariate Cox regression analyses of overall survival

		Univariate analysis			Multivariate model adjusted for age		
	*n*	5-year rate	95% CI	p**	Hazard ratio	95% CI	*p*
All	2158	81.3	79.7–82.9				
Age (years							
< 65	1079	88.3	86.3–90.3				
≥ 65	1079	74.4	71.9–76.9	< 0.001			
Sex							
Male	1351	80.2	78.0–82.4		1.0		
Female	807	83.2	80.7–85.7	0.001	0.8	0.7–0.9	0.002
ASA*							
ASA I-II	1498	85.2	83.4–87.0	85.2	1.0		
ASA III-IV	336	63.6	58.3–68.9	63.6	1.9	1.6–2.3	< 0.001
Tumour site							
Colon	990	82.5	80.1–84.9				
Rectum	1168	80.3	77.9–82.7	0.306			
Stage (UICC)							
Stage I	573	88.2	85.5–90.9		1.0		
Stage II	535	81.7	78.4–85.0		1.2	1.0–1.5	0.120
Stage III	500	70.9	66.8–75.0		1.7	1.3–2.0	< 0.001
Stage y0	73	98.6	95.9–100		0.7	0.4–1.3	0.290
Stage yI	164	94.4	90.9–97.9		0.9	0.6–1.3	0.504
Stage yII	146	74.6	67.3–81.9		1.8	1.3–2.4	< 0.001
Stage yIII	167	73.5	66.8–80.2	< 0.001	2.3	1.7–3.0	< 0.001
Clavien-Dindo classification							
0	1691	83.5	81.7–85.3		1.0		
I	141	76.2	69.1–93.3		1.6	1.2–2.1	< 0.001
II	162	72.7	65.8–79.6		1.5	1.2–1.9	0.001
III	112	76.5	68.7–84.3		1.4	1.1–2.0	0.022
IV	52	63.1	50.0–76.2	< 0.001	1.9	1.3–2.7	0.001

*ASA* American Society of Anesthesiologists Classification; *ASA missing in 324 patients

**Log-rank test

## Results over time

If the study time is divided and the two periods, 1995–2004 (*n* = 1097) and 2005–2014 (*n* = 1061), are compared, we found fewer POCs in the second period (23.2% vs 20.0%; *p* = 0.066), in particular a decrease in POCs of CDC grade IV was observed (3.4% vs 1.4%; *p* = 0.003). With regard to long-term outcome, the 5-year locoregional recurrence rate decreased significantly from 7.3% (95%CI 5.7–8.9) to 3.8% (95% CI 2.6–5.0; *p* = 0.006), while improvements in distant metastasis (18.5% vs 14.8%; *p* = 0.082), disease-free survival (70.9% vs 76.0%; *p* = 0.092) and overall survival (79.5% vs 83.3%; *p* = 0.239) did not reach significance.

## Discussion

The present study shows that men, ASA III–IV patients, patients with rectal carcinomas, patients who underwent abdominoperineal excisions and patients who underwent multivisceral resections have more POCs and higher CDC grades than other patients. In investigating the influence of postoperative complications on long-term prognosis, a worse prognosis was found in patients with postoperative complications, especially in patients with high CDC grades. In particular, CDC grade III patients were associated with an increased risk for locoregional recurrences and distant metastases. This corresponds to the recurrence rates in the study by Duraes et al. [6], in which CDC grade III patients also had the worst recurrence rate. In addition, patients with CDC grade IV POCs showed significantly worse disease-free and overall survival rates, which is also consistent with the results of the study mentioned above. Postoperative complications usually depend on the extent of the surgical intervention. Sigmoid resection of a small tumour is typically associated with fewer and less serious complications than total pelvic exenteration [17].

Most patients with CDC grade III complications had surgical complications. In our series, 89% (97 of 112 patients) of the patients with CDC grade III POCs had a surgical complication. These patients required endoscopic, radiological or surgical interventions. Anastomotic leak is a typical example of a CDC grade III complication frequently associated with increased rates of locoregional recurrences and even poor overall survival [2, 3]. This can be explained by exfoliated cancer cells that remain after tumour resection, which may lead to local recurrence induced by a pelvic infection with systemic inflammatory and immunomodulatory responses primarily caused by the anastomotic leak.

CDC grade IV complications are life-threatening and require the patient to be admitted to an intermediate or intensive care unit. The results of the present study show that these patients had the worst disease-free and overall survival rates compared to other patients. It should be noted that these severe complications are not necessarily surgical complications. In the present study, 40% of CDC IV patients had non-surgical complications. Preoperative comorbidities, such as chronic heart failure, chronic obstructive pulmonary disease (COPD) and renal failure, represent a particular risk for non-surgical complications. These comorbidities can lead to single- or multi-organ dysfunction after colorectal surgery, which affects survival outcomes regardless of the original oncological prognosis.

Artinyan et al. [18] showed that postoperative complications after the resection of colorectal carcinomas are an independent risk factor for poor long-term survival. In particular, patients with infectious complications, such as deep and superficial surgical site infections, pneumonia or urinary tract infections, have been associated with increased rates of locoregional recurrence and decreased long-term prognosis. This association is especially impressive in view of the fact that the affected patients in the study were young and had few preoperative comorbidities. In our study, severe infectious complications such as peritonitis, colon necrosis or pneumonia were found in 46% of the patients with CDC IV POCs, who had decreased disease-free and overall survival rates. Previous studies have shown that C-reactive protein (CRP) can predict the occurrence of complications [19] and even the severity of postoperative complications in colorectal cancer [20]. Among patients with anastomotic leaks, patients who required reoperations were found to have worse overall survival compared to those for whom conservative treatment was sufficient [21].

The ASA classification, which assesses the preoperative status of the patient, is an independent factor for morbidity and mortality. An older age is frequently associated with more comorbidities and thus with an increased ASA classification. The present study showed that patients with more serious comorbidities (ASA III or IV classification) were associated with more frequent complications and a higher CDC grade. This is reflected in the worse long-term prognosis with shorter disease-free and overall survival when patients with comorbidities do not recover properly from the complications of surgery. Patients who are more ill are more likely to have a higher complication rate and thus a lower survival rate. As a complication-free course is important for the overall prognosis, good preoperative preparation is essential, particularly for older and high-risk patients. In addition to ensuring appropriately treated comorbidities, such as good control of diabetes and hypertension, optimal preoperative and postoperative nutrition and physical activity play an important role. Malietzis et al. found an association between muscle mass and postoperative morbidity and mortality. They identified myopenia as an independent risk factor for disease-free and overall survival in patients with colorectal cancer [22]. It has also been found that physical exercise programs improve the symptoms of the side effects of chemotherapy and thus enable an increase in the completion rate of chemotherapy in patients with colorectal carcinoma [23], which ultimately affects disease-free and overall survival.

Patients with complications have an increased risk of not receiving adequate adjuvant therapy. The lack of receiving adjuvant therapy postoperatively is known to increase metastasis rate and to decrease survival. In addition, if adjuvant treatment is indicated after surgery, it should start on time; otherwise, the prognosis will deteriorate [24]. According to the German Evidenced-based Guideline for Colorectal Cancer, colon cancer patients should start adjuvant chemotherapy within 8 weeks [15]. This is supported by a complication-free postoperative course, while severe complications may prevent or delay the receipt of adjuvant chemotherapy [3]. In a systematic review and meta-analysis, Biagi et al. identified an inverse correlation between the time to adjuvant chemotherapy and survival [25]. This is supported by the study by Bayraktar et al. [26]. These results showed that starting adjuvant treatment in stage II and III colon carcinoma patients more than 60 days after surgery worsens the prognosis. Lima et al. [27] found no difference in survival between patients who started adjuvant treatment for stage III colon cancer within 8 weeks or 8 to 12 weeks. However, patients who received adjuvant chemotherapy after 12 to 16 weeks had worse survival, and those who started after more than 16 weeks had a prognosis similar to that of patients who did not receive adjuvant treatment. Interestingly, the group of patients who received adjuvant chemotherapy more than 12 weeks after surgery were associated with a poor socioeconomic level and more comorbidities. Nachiappan et al. [28] found that reoperation does not worsen survival if adjuvant chemotherapy can still begin without delay.

Nevertheless, the anatomical extent of the tumour (TNM classification) and presence of residual tumour (R classification) are still the most important prognostic factors for the oncologic outcome. High-quality surgery, as part of a multidisciplinary care strategy, is essential for a complication-free postoperative course and a favourable long-term outcome. The increasing specialization in colorectal surgery contributes to this conclusion [29].

Our study has some limitations, particularly the single-centre design and its retrospective nature. During the long study time of 20 years, treatment protocols have changed, and multimodal treatment has gained importance. For example, the administration of adjuvant chemotherapy for stage III colon cancer nearly doubled from 44% in 1995–1999 to 79% in 2010–2014.

In conclusion, this study identified postoperative complications as an inverse prognostic factor in colorectal cancer with a strong relationship to the Clavien-Dindo classification. It emphasizes the importance of a complication-free postoperative course, not only in view of the immediate postoperative problems and costs but also for the long-term course. This relevance should be known to the entire treating and caring team during the preoperative preparation, the actual surgery and in the postoperative course. It also should be discussed with the patient, so that he/she will definitely attend all follow-up examinations.
